# Long-term manure amendments reduced soil aggregate stability via redistribution of the glomalin-related soil protein in macroaggregates

**DOI:** 10.1038/srep14687

**Published:** 2015-10-01

**Authors:** Hongtu Xie, Jianwei Li, Bin Zhang, Lianfeng Wang, Jingkuan Wang, Hongbo He, Xudong Zhang

**Affiliations:** 1 State Key Laboratory of Forest and Soil Ecology, Institute of Applied Ecology, Chinese Academy of Sciences, Shenyang, 110164, China; 2 Department of Agriculture and Environmental Sciences, Tennessee State University, Nashville, TN 37209, USA; 3 College of Land & Environment, Shenyang Agricultural University, Shenyang, 110866, China; 4 College of Environmental and Chemical Engineering, Dalian Jiaotong University, Dalian, 116028, China; 5 National Field Research Station of Agroecosystems, Institute of Applied Ecology, Chinese Academy of Sciences, Shenyang, 110016, China

## Abstract

Glomalin-related soil protein (GRSP) contributes to the formation and maintenance of soil aggregates, it is however remains unclear whether long-term intensive manure amendments alter soil aggregates stability and whether GRSP regulates these changes. Based on a three-decade long fertilization experiment in northeast China, this study examined the impact of long-term manure input on soil organic carbon (SOC), total and easily extractable GRSP (GRSP_t_ and GRSP_e_) and their respective allocations in four soil aggregates (>2000 μm; 2000–250 μm; 250–53 μm; and <53 μm). The treatments include no fertilization (CK), low and high manure amendment (M1, M2), chemical nitrogen, phosphorus and potassium fertilizers (NPK), and combined manure and chemical fertilizers (NPKM1, NPKM2). Though SOC, GRSP_e_ and GRSP_t_ in soil and SOC in each aggregate generally increased with increasing manure input, GRSP_t_ and GRSP_e_ in each aggregate showed varying changes with manure input. Both GRSP in macroaggregates (2000–250 μm) were significantly higher under low manure input, a pattern consistent with changes in soil aggregate stability. Constituting 38~49% of soil mass, macroaggregates likely contributed to the nonlinear changes of aggregate stability under manure amendments. The regulatory process of GRSP allocations in soil aggregates has important implications for manure management under intensive agriculture.

Glomalin is a glycoproteinaceous substance that is produced by arbuscular mycorrhizal fungi (AMF)^1^,^2^. Glomalin is usually quantified in soils as glomalin-related soil protein (GRSP)^2^,^3^,^4^,^5^, which is a component of the hyphal walls of AMF and is likely released into the soil after death^6^; thus, GRSP subsequently contributes to the linkage between soil particles and the stabilization of aggregates. Positive correlations between the GRSP concentration and the amount of water-stable aggregates have been documented^2^,^3^,^7^. In addition, glomalin is relatively recalcitrant and has a long residence time in the soil to contribute to stable carbon storage^8^,^9^; microbial-derived carbon inputs to soils are being recognized as increasingly important in the long-term storage of carbon and nitrogen^10^.

Despite recalcitrant features, GRSP can be sensitive to various agricultural management practices, such as tillage^11^,^12^,^13^,^14^, cropping treatments^11^,^15^, and land use change^16^,^17^,^18^. Chemical and organic fertilizations are common practices and play a key role in maintaining long-term agricultural production; however, the effects of different types of fertilization on the changes in glomalin concentrations have received very limited attention. For example, long-term fertilization, especially amendments with manure and straw, increase soil GRSP accumulation^19^,^20^. However, the effects of different amounts of manure and mineral fertilizer and their interactions on GRSP dynamics have not been elucidated.

Aggregates are composed of primary mineral particles and organic binding agents^21^. Therein, arbuscular mycorrhizal fungi produce large amounts of insoluble glycoprotein, glomalin and polysaccharides, which contribute to aggregate stability^2^,^4^. Furthermore, many studies have examined the glomalin concentration in soil aggregates, but most studies only focused on the 1000- to 2000-μm aggregates^2^,^22^. It was found that approximately 20% of GRSP remained in the fine fraction (<53 μm)^12^. Other study indicated that tillage reduced the GRSP content in all of the aggregate classes (2000–1000 μm, 1000–500 μm and 250 μm)^11^. These results collectively suggest a possible strong correlation of glomalin with aggregate stability^2^. However, the underlying mechanism of this tight association between GRSP and aggregate stabilization remains poorly investigated. Furthermore, understanding factors controlling GRSP production such as fungal community composition, fungal physiology, and cell biology aspects as well as soil biota, soil physicochemical characteristics, and fungus–host plant species combinations will elucidate soil aggregation in crop production systems^23^.

Long-term experiments provide a realistic and effective means for obtaining valuable information that is required to maintain the soil quality and health by determining changes in the soil properties and processes^24^,^25^. Soil fertility degradation has long been a major concern in China due to the replacement of organic fertilizers by chemical fertilizers^26^. To monitor changes in soil fertility, a number of long-term experiments were initiated in typical agricultural regions in China in the 1980’s with the application of chemical fertilizer, organic manure alone or both in combination^26^. One of these experiments was set up in a brown soil region located in Liaohe Plain to explore the effect of long-term fertilization on the soil properties and crop yield^27^. Soil aggregate formation and stability are key variables for investigation; however, the role of GRSP on soil aggregates during long-term fertilization experiments has received little attention in this carbon-rich and high-productivity agricultural region, which hindered our understandings of how management practices alter GRSP concentrations in soil aggregates and how to maintain soil aggregate stability, fertility and productivity under different fertilization practices.

Based on a three-decade long fertilization experiment in a typical brown soil in Northeast China, we collected surface soil samples (0–20 cm), quantified soil aggregate and GRSP concentrations, and compared the long-term dynamics of GRSP in different soil aggregate sizes under a suite of long-term continuous chemical and organic fertilizer treatments. The objectives of this study were to examine the effects of the long-term application of mineral and organic fertilizers alone or in combination on the concentration and allocation of GRSP in different aggregate classes. This study is expected to clarify on the relationship between GRSP in different soil aggregate sizes and aggregate stability for the sake of optimal management practices in this region.

## Results

### Long-term manure inputs on aggregate distribution and stability

The proportional distribution of aggregates in soil generally followed a descending order in each fertilization regime: small macroaggregate > microaggregate > silt + clay > macroaggregate (Table 1). The small macroaggregate comprised the largest proportion of the soil (35.9–49.1%), and the large macroaggregate accounted for 11.4% to 18.9%. Different fertilization regimes altered the aggregate percentages in the soil. Organic manure application alone or in combination with fertilizer (M1, M1NPK, M2 and M2NPK) increased the proportion of the small macroaggregate (*P* < 0.1) and decreased the percentages of microaggregate and silt + clay (*P* < 0.1) compared to CK and NPK. High amounts of organic manure input (M2, M2NPK) tended to diminish the proportion of large macroaggregate compared to the other treatments. Furthermore, NPK did not change the distribution of aggregates compared to CK.

Compared to CK, NPK, M2 and M2NPK, low-manure application (M1 and M1NPK) increased significantly MWD by approximately 20% (*P* < 0.05). Compared to CK, NPK, M2 and M2NPK decreased MWD by 5%, 5% and 11%, respectively, but these effects are not statistically significant.

### Long-term manure inputs on the SOC in bulk soil and aggregates

Generally, organic manure application significantly increased the SOC concentration in bulk soil (P < 0.05). There were significant differences in the SOC between CK and all fertilization treatments. Compared to CK, SOC increased by 25.9% under NPK and by 30.8% to 47.7% under organic manure treatment alone or combined manure and chemical fertilizers (M2NPK > M1NPK > M2 > M1 > NPK > CK) (Table 2).

**Figure 1:**
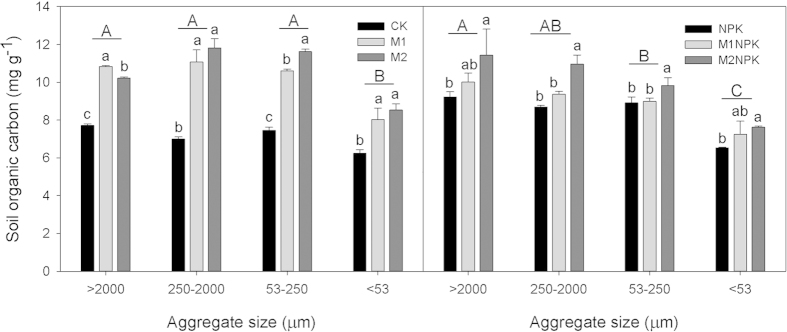
SOC content (mg g^−1^) in soil aggregates under different fertilization treatments in Shenyang Agricultural University experiment in Liaoning, China. The different lowercase letters represent significant fertilization effect within each aggregate size (*P* < 0.05), and the different uppercase letters denote significant effect of aggregate sizes (*P* < 0.05). Error bars denote standard deviations (n = 3). The abbreviations of fertilization treatments are the same as presented in Table 1.

Across all fertilization treatments, SOC concentration in macroaggregates and microaggregates showed little difference, but SOC concentration in sum of macroaggregates and microaggregates (>53 μm) and silt + clay fractions (<53 μm) were significantly different (*P* < 0.05, Fig. 1). Fertilizer application significantly altered SOC concentration in each aggregate fraction with a descending order as M2, M2NPK > M1, M1NPK > NPK > CK. Compared to CK, SOC showed a significantly greater increase under organic manure application alone or in combination with chemical fertilizers than chemical fertilizers alone (*P* < 0.05, Fig. 1).

### Long-term manure inputs on the GRSP in soil aggregates

Similar to the patterns of SOC changes under fertilization, long-term fertilization significantly increased GRSP_t_ and GRSP_e_ concentrations in bulk soil (M2NPK, M2, M1NPK > M1 > NPK > CK) (Table 2). The M1 treatments showed a lower GRSP_e_ content and higher amount of GRSP_t_ compared with NPK treatments. Chemical fertilizer treatment alone led to a greater ratio of GRSP_e_/GRSP_t_ (NPK > M2, M1NPK, M2NPK). In general, high manure amendments induced significantly greater GRSP_t_/SOC ratios than other treatments (M2NPK, M2, M1NPK > M1, NPK, CK).

**Figure 2:**
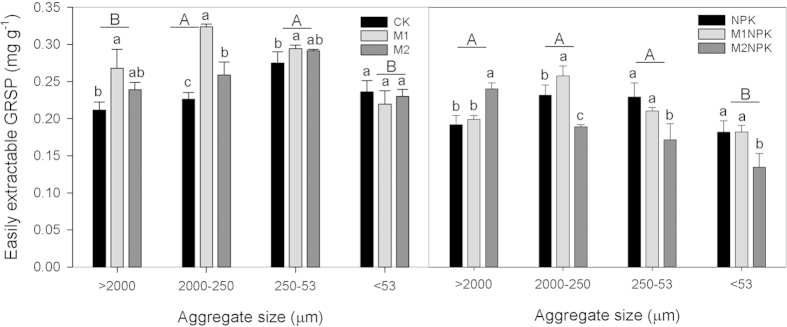
GRSP_e_ content (mg g^−1^) in soil aggregates under different fertilization treatments in Shenyang Agricultural University experiment in Liaoning, China. The different lowercase letters represent significant fertilization effect within each aggregate size (*P* < 0.05), and the different uppercase letters denote significant effect of aggregate sizes (*P* < 0.05). Error bars denote standard deviations (n = 3). The abbreviations of fertilization treatments are the same as presented in Table 1.

**Figure 3:**
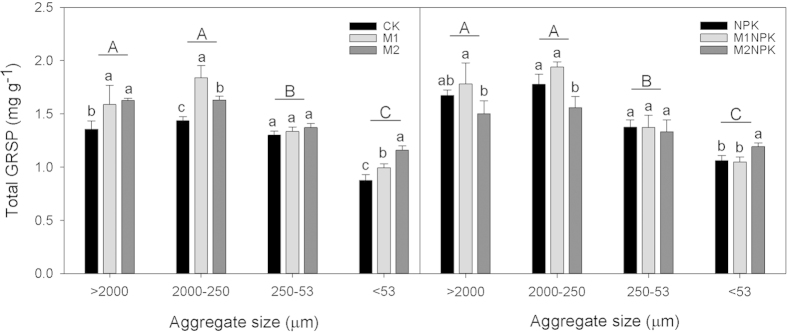
GRSP_t_ content (mg g^−1^) in soil aggregates under different fertilization treatments in Shenyang Agricultural University experiment in Liaoning, China. The different lowercase letters represent significant fertilization effect within each aggregate size (*P* < 0.05), and the different uppercase letters denote significant effect of aggregate sizes (*P* < 0.05). Error bars denote standard deviations (n = 3). The abbreviations of fertilization treatments are the same as presented in Table 1.

Long-term fertilization significantly increased SOC allocation within each aggregate than no fertilization (M2NPK > NPK > CK, Fig. 1), whereas, fertilization effects on GRSP allocations were highly variable with different aggregate sizes. The above mentioned pattern of SOC allocation was true for GRSP_e_ in macroaggregates only (Fig. 2) and true for GRSP_t_ in silt + clay fraction only (Fig. 3).

## Discussion

### Manure amendments on soil aggregate content and stability

Generally, the combined application of organic manure and fertilizer increased the proportions of small macroaggregate and decreased the proportions of microaggregate in the soil^28^,^29^. In our study, changes in the proportions of soil aggregates varied with different fertilization treatments. For instance, NPK treatment decreased the proportion of small macroaggregates, but manure or manure plus chemical fertilizers increased the same size aggregate. In contrast, NPK increased the proportion of microaggregates, but manure or manure plus chemical fertilizers decreased the same size aggregate.

As a measure of aggregate stability, mean weight diameter (MWD) has been used to assess manure amendments on soil aggregate stability. A recent study indicated that as compared to CK, 2-year long-term manure addition (60 Mg·hm^−2^) increased the MWD at the 0–5cm layer in soil type of Typic Haplargids^30^, whereas, this study showed that three-decade high manure application decreased the MWD and consequently resulted in lower aggregate stability as compared to CK. This contradiction may be due to the accumulation of large macroaggregates that were derived from different sources of manure materials. In this study, a very high amount of manure amendment provided POC^31^, which coated macroaggregates. Long-term manure applications with a large amount of organic input increased the macroaggregate dispersion and thus resulted in a decrease in aggregate stability^32^. However, other studies suggest the MWD increased after 1 year manure application and decreased after 5 years and increased again after 12 years, indicating that long-term manure application (1 Mg·hm^−2^) increase soil aggregate stability^33^. In addition, there is unclear relationships between aggregate stability and rates of organic input by analyzing the literatures due to different factors such as the quality, quantity and timing of organic matter addition^34^. In particular, few studies offered mechanistic understanding of manure amendments and soil aggregate stability. In the following discussions, this study addressed the fertilization effect on SOC, GRSP allocations in different soil aggregates and particularly examined the possible mechanisms of GRSP redistribution driving the change of soil aggregate stability.

### Manure amendments on SOC allocation in soil aggregates

Manure application has long been recognized as an effective way to increase SOC content^29^,^32^,^35^,^36^,^37^,^40^. This study showed that manure application alone or mixed with chemical fertilizers significantly improved the total SOC content compared to that of CK and NPK. On the other hand, the application of NPK fertilizers also significantly increased the SOC content compared to that of CK, which is driven by greater yield and biomass return to the SOC pool^37^. The reason for this result is that the manure input directly increased the soil organic matter content and induced the additional input of organic material to soils due to higher crop productivity under fertilization^37^,^38^,^39^.

This study further demonstrated that fertilizer application significantly increased the SOC content in every aggregate fraction compared with CK. Especially, manure and manure combined with fertilizer enhanced SOC concentration in macroaggregates and microaggregates (*P* < 0.05), indicating that the manure-derived C was more preferentially accumulated in these aggregate fractions^37^. For all of the fertilization treatments, the lowest SOC contents were found in the silt + clay fraction. A similar observation was reported through long-term fertilization on a Mollisol^36^. These observations are most likely associated with low or no binding capacity of SOC by free silt particles^36^, and also the limited protection of SOC by silt and clay^41^. Overall, this study revealed that manure application alone or in combination with chemical fertilizer increased SOC through their effects on the formation of macroaggregates. This result is consistent with a former study that showed that animal manure application increased SOC and consequently the formation of macroaggregates^38^,^41^,^42^. Given the relatively stable soil mineralization rate indicated in these studies, we speculate that the SOC change is directly related to C input from manure^38^,^43^. In addition, the low temperature in winter restricts the decomposition of manure^38^.

### Manure amendments on GRSP in bulk soil

As an important component in SOC, the concentrations of both GRSP_e_ and GRSP_t_ were enhanced significantly under treatments with manure input (*P* < 0.05; Table 2), which echoed with several recent studies^2^,^19^,^20^,^44^,^45^,^46^. The main reason may lie in the release of the growth-stimulating substances due to increased soil biological activities and nutrients from organic manure^6^,^19^,^46^. On the other hand, the relatively low amount of GRSP_t_ under NPK treatment compared with manure amendment treatments most likely due to the inhibition of AMF development by chemical fertilizers^20^,^46^. However, the greater GRSP_e_/GRSP_t_ under NPK than manure amendment treatments could be caused by the immediate and pronounced effect of nutrient availability on GRSP_e_ productions via part of AMF groups under NPK treatment. It thus remains to be further explored whether only some species of AMF development are more sensitive to chemical nutrient input than other species.

The SOC content is often a good predictor of GRSP^9^. In our study, the GRSP_t_/SOC changed from 0.18 to 0.21 when manure was applied either with chemical fertilizers or at a higher input rate. This pattern of change under fertilization is much narrower in quantity in comparison to former studies in agroecosystem (0.14 to 0.27)^44^ and in different land use types (0.21 to 0.29)^47^. This current result also showed a positive correlation between the GRSP and SOC content. This relationship was affected by land use type and soil type such as in pastures^48^, Mediterranean steppes^17^, North American soils^49^ and a semiarid rangeland^50^. The exceptions to this trend are the Costa Rican study^51^ and a strong acid soil result from the narrow range of SOC^52^. In our study, a high-manure amendment did not increase the contribution of GRSP_t_ to SOC due to parallel increases in both GRSP_t_ and SOC. This result clearly indicates that GRSP, as an important component of soil organic matter and binding agents^53^, can contribute to soil carbon sequestration under long-term manure amendments^54^.

### Manure amendments on GRSP allocation in soil aggregates

**Figure 4:**
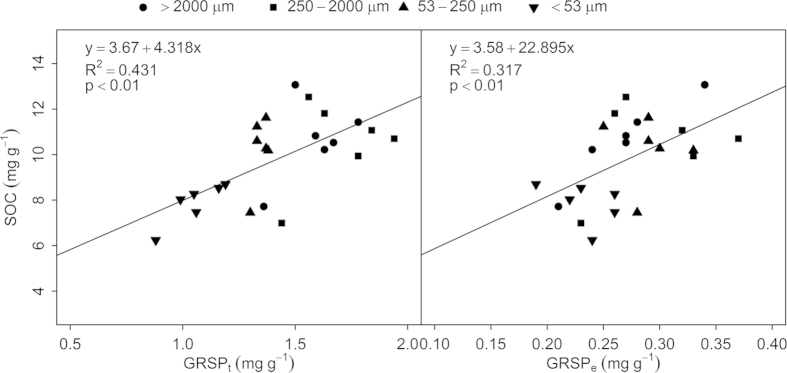
The regression between SOC and GRSP across different aggregate fractions.

As we revealed above, three-decade-long manure amendments combined with mineral fertilizer application significantly increased soil aggregate stability, while the underlying mechanisms are not well identified. This study found that long-term manure amendment combined with chemical fertilizer application significantly increased the GRSP content in all soil aggregate fractions except for microaggregates (Fig. 1). In particular, intermediate amount of manure input in addition to chemical fertilizers (M1NPK) increased the content of macroaggregates and the overall soil aggregate stability (Table 1). These results suggest that glomalin accumulation influenced soil stability via its redistribution in macroaggregates under long-term fertilization. In all aggregate fractions, the relationship of GRSP and SOC showed significant trend (Fig. 4. R^2^ = 0.431 and R^2^ = 0.317, *P* < 0.01). The accumulation of GRSP in total SOC is possibly attributed to the positive role of AMF in glomalin production at presence of long-term and relatively large amount of manure amendments in soils^6^,^19^,^55^.

Although the application of organic manure positively affected the accumulation of GRSP in aggregates, the highest amount of organic manure did not result in the highest concentration of either GRSP_e_ or GRSP_t_. In fact, the contents of GRSP_e_ and GRSP_t_ in the small macroaggregates were significantly lower under high manure input treatments than low manure input treatments. Thus, a high amount of organic manure amendment exceeding a certain threshold may otherwise reduce GRSP content and then decrease the soil aggregate stability by altering GRSP allocations to macroaggregates (Table 1).

## Methods

### Site description and soil sampling

This study was conducted on a long-term fertilization trial that was initiated in April 1979 at the Experimental Station of Shenyang Agricultural University (41°48´N, 123°33’E) in Liaoning Province, China. The annual mean temperature ranged from 7.0 to 8.1 °C, the annual mean precipitation ranged from 574 to 684 mm, and the average frost-free period was 147 to 164 days in the past 30 years. The soil is a Hapli-Udic Cambisol (FAO Classification). Prior to this experiment, the concentration of SOC was 9.2 g kg^−1^, the total nitrogen concentration was 0.8 g kg^−1^, and the soil pH was 6.5 (soil:water = 1:2.5) at the top 20-cm depth.

The current long-term experiment consists of a randomized complete block design with three blocks and eighteen fertilization treatments. The area of one individual plot was 160 m^2^. The following six treatments were included in this study: (1) no fertilizer (CK); (2) chemical nitrogen, phosphorus and potassium fertilizers (NPK); (3) low manure amendment (M1); (4) combination of M1 and NPK (M1NPK); (5) high manure amendment (M2); and (6) combination of M2 and NPK (M2NPK). M1 and M2 refer to composted pig manure applied at the rate of 13.5 and 27 Mg hm^−2^ yr^−1^ (organic matter 119.6 g kg^−1^; total N 5.8 g kg^−1^; P 3.6 g kg^−1^; K 9.0 g kg^−1^), respectively; NPK denotes chemical N (urea), P (multiple superphosphate), and K (potassium sulfate) fertilizers added at the rate of 135, 29, and 56 kg hm^−2^ yr^−1^, respectively. The plots have a cropping system of monoculture maize (*Zea mays* L.). Maize was planted in late April and harvested in late September every year. The mineral fertilizers were evenly distributed on the soil surface by hand and immediately incorporated into the soil by tillage before sowing in April. Dry-composted pig manure was spread over the soil surface after harvesting in October. Surface soil samples (0–20 cm) from each plot were collected in October 2008 prior to manure amendment. The field-moist soil samples were gently broken apart, sieved to pass through a 5-mm sieve, and air-dried for physiochemical analysis.

### Water-stable aggregate fractionation

Four aggregate-size fractions were separated by wet sieving according to Elliott’s method^56^. and they were named as large macroaggregate (>2000 μm), small macroaggregate (2000–250 μm), microaggregate (250–53 μm), and silt + clay fraction (<53 μm). Briefly, 100 g of soil (oven-dry equivalent weight) was submerged in deionized water on top of a 2000-μm sieve overnight at room temperature. The aggregates were then separated by moving the sieve up and down 50 times over a period of 2 min. Then, the intact aggregates were washed off the sieve and collected in an aluminum pan. The remaining soil slurry was passed through the 250- and 53-μm sieves, while the sieving procedure described above was repeated. The silt + clay fraction was separated by centrifuging (2500 × g, 10 min) the soil suspension that passed through the 53-μm sieve. After being oven dried at 50 °C, the four classes of aggregates were weighed and stored at room temperature for future use. The mean weight diameter (MWD) represents fraction of the sample on the sieve times mean intersieve aperture and was used to indicate the soil aggregate stability^57^. The total carbon in the soil aggregates was determined by dry combustion using an element analyzer Vario Elementar III (Elementar Analysensysteme GmbH, Hanau, Germany). Because these soil samples were free of carbonates, the total carbon content was equivalent to the soil organic carbon content.

### GRSP extraction and determination

GRSP was extracted according to the procedures described by Wright and Upadhyaya^2^. Briefly, extractable GRSP (GRSP_e_) was extracted from 1 g of 2-mm-sieved soil with 8 ml of a 20 mM citrate solution at pH 7.0 by autoclaving at 121 °C for 30 min, and then the supernatant was removed by centrifugation at 10,000 × g for 5 min. The total GRSP (GRSP_t_) was extracted with 8 ml of 50 mM citrate solution at pH 8.0 by autoclaving at 121 °C for 60 min, then centrifuged at 10,000 × g for 5 min to remove the supernatant. After each cycle, the sodium citrate was replenished for the extraction again until the GRSP content of supernatant was above the detection limits (ca. 2 mg ml^−1^). The supernatant was decanted and stored at 4 °C until being analyzed. The protein content was determined by the Bradford assay^58^ using bovine serum albumin as a standard.

### Statistical analysis

The significant difference of the effects of aggregate size and the fertilization treatment on the SOC, GRSP_t_, GRSP_e_ and GRSP_t_/SOC were assessed using one-way analyses of variance (ANOVA). Post hoc analyses were conducted using LSD tests. A statistical significance was set at P < 0.05 or P < 0.1. All of the statistical analyses were implemented in the R program^59^.

## Additional Information

**How to cite this article**: Xie, H. *et al.* Long-term manure amendments reduced soil aggregate stability via redistribution of the glomalin-related soil protein in macroaggregates. *Sci. Rep.* **5**, 14687; doi: 10.1038/srep14687 (2015).

## Figures and Tables

**Table 1:** Mean (±SD) proportions of four aggregates in soil (%)under long-term fertilization in Liaoning, China.

Treatment†	Aggregate distribution (%)				MWD(mm)
	Large macroaggregate (>2000 μm)	Small macroaggregate (2000–250 μm)	Microaggregate (250–53 μm)	Silt + clay(<53 μm)	
CK	14.8 ± 2.4ab‡	37.9 ± 2.0b	26.9 ± 1.1ab	16.8 ± 3.5ab	0.99 ± 0.11b
NPK	13.6 ± 1.7abc	35.9 ± 5.4b	29.6 ± 1.3a	18.4 ± 2.5a	0.93 ± 0.12b
M1	18.9 ± 5.1a	44.7 ± 4.0ab	21.6 ± 4.7b	11.6 ± 1.4b	1.20 ± 0.23a
M1NPK	17.4 ± 2.3ab	46.3 ± 1.7ab	20.6 ± 1.0b	12.8 ± 0.6ab	1.17 ± 0.10a
M2	10.8 ± 0.4c	49.1 ± 4.7a	26.2 ± 3.0ab	12.3 ± 2.1b	0.88 ± 0.07b
M2NPK	11.4 ± 1.1bc	44.4 ± 3.8ab	26.8 ± 1.6ab	15.2 ± 1.4ab	0.94 ± 0.09b

Soil samples were collected in 2008.

^†^CK, no fertilizer; NPK, mineral fertilizers; M1 and M2, organic manure applied at lower and higher level, respectively; M1NPK, combination of M1 and NPK; M2NPK, combination of M2 and NPK.

^‡^Different letters within each column indicate significant difference between fertilization treatments at 0.05 (n = 3).

**Table 2:** Mean (±SD) concentrationsof GRSP_t_, GRSP_e_ and SOC (mg g^−1^) in bulk soil in different fertilization treatments in Shenyang Agricultural University experiment in Liaoning, China.

Treatments	GRSP_e_	GRSP_t_	SOC	GRSP_e_/GRSP_t_	GRSP_t_/SOC
	mg g^−1^				
CK	0.24 ± 0.02c	1.41 ± 0.08c	7.95 ± 0.15d	0.17ab	0.18b
NPK	0.35 ± 0.05ab	1.80 ± 0.20b	10.01 ± 0.09c	0.20a	0.18b
M1	0.30 ± 0.01b	1.86 ± 0.06b	10.40 ± 0.10b	0.16ab	0.18b
M1NPK	0.37 ± 0.02a	2.46 ± 0.07a	11.65 ± 0.25a	0.15b	0.21a
M2	0.38 ± 0.02a	2.46 ± 0.13a	11.55 ± 0.15a	0.16b	0.21a
M2NPK	0.38 ± 0.01a	2.45 ± 0.06a	11.75 ± 0.05a	0.16b	0.21a

Soil samples were collected in 2008.
